# Exploring barriers to IVR surveys and the effectiveness of human follow-up calls: insights from a mixed methods study in Uganda

**DOI:** 10.1093/oodh/oqaf017

**Published:** 2025-08-16

**Authors:** Raymond Tweheyo, Dustin Gibson, Helen Kuo, Joe Ali, Michelle Kaufman, Andres Vecino Ortiz, Elizeus Rutebemberwa

**Affiliations:** Department of Health Policy Planning and Management, Makerere University College of Health Sciences, School of Public Health, Mulago Hill-Road. P.O.Box, 7072, Kampala, Uganda; Department of International Health, Johns Hopkins University Bloomberg School of Public Health, 615 N. Wolfe Street, Baltimore, MD 21205, United States; Department of International Health, Johns Hopkins University Bloomberg School of Public Health, 615 N. Wolfe Street, Baltimore, MD 21205, United States; Department of International Health, Johns Hopkins University Bloomberg School of Public Health, 615 N. Wolfe Street, Baltimore, MD 21205, United States; Department of Population, Family, and Reproductive Health, Johns Hopkins University Bloomberg School of Public Health, 615 N. Wolfe Street, Baltimore, MD 21205, United States; Department of International Health, Johns Hopkins University Bloomberg School of Public Health, 615 N. Wolfe Street, Baltimore, MD 21205, United States; Department of Health Policy Planning and Management, Makerere University College of Health Sciences, School of Public Health, Mulago Hill-Road. P.O.Box, 7072, Kampala, Uganda

**Keywords:** interactive voice response, mobile phone surveys, computer-assisted telephone interview, non-response, mixed methods, Uganda

## Abstract

**Background:**

This study explored reasons why respondents neither initiate nor complete an interactive voice response (IVR) survey and whether call-backs by a human can increase subsequent IVR survey participation.

**Methods:**

We conducted a mixed methods study. Using random digit dialing (RDD), participants were sent an IVR survey (IVR-RDD) to their mobile phone. Participants from the IVR-RDD who either did not pick the phone or terminated the survey within two questions were contacted for a computer-assisted telephone interview (CATI) survey to assess reasons for non-participation. Following CATI completion, a similar IVR survey was sent (post-CATI IVR). Descriptive statistics and adjusted logistic regression models were conducted to assess differences in survey outcomes between the IVR-RDD and the post-CATI IVR groups.

**Results:**

A total of 23 288 IVR-RDD, 9740 CATI and 1000 post CATI IVR calls were made to yield 1.9%, 11.8% and 44.9% response rates, respectively. The most common reasons for non-response or drop-off to the IVR-RDD were being busy, misunderstanding IVR instructions and mistrust of the IVR caller. Compared to the IVR-RDD, the post-CATI IVR increased both contact rate, from [(2.9%; 669/23062) to (7.74%; 1758/22704); adjusted odds ratio (AOR) 2.81, 95% confidence interval (95%CI) 2.56, 3.08, *P* < 0.001] and response rate, from [(2.25%; 518/23062) to 4.54% (1031/22704); AOR 2.07, 95%CI 1.86, 2.30, *P* < 0.001], but no impact on the cooperation rate.

**Conclusions:**

Understanding reasons for survey non-response can allow for interventions to improve survey response. Introducing a human interviewer to those who did not complete the IVR survey improves subsequent IVR survey participation rates.

## INTRODUCTION

The coverage of mobile phone ownership in low- and middle-income countries (LMICs) has approached saturation levels and is steadily closing the gap that exists with high-income countries (HIC); offering an opportunity for reaching communities with mobile phone surveys. For example, in 2020, there were 99/100 vs 133/100 active mobile phone subscriptions per 100 people in LMICs and HICs, respectively [1]. With an increasing burden of non-communicable diseases (NCDs), both in Uganda and globally [2], population based surveys, like the World Health Organization (WHO)’s Stepwise approach to NCD risk factor surveillance (STEPS) are an important tool in understanding and curbing their rise. However, these surveys are expensive to conduct due to personnel and transportation requirements and are typically deployed every 5 years [3]. As such, alternative survey approaches, such as interviewing respondents over their mobile phone through interactive voice response (IVR) surveys [4, 5], are needed.

IVR systems are defined as interfaces that replace the need for a live operator, by allowing participants to respond to pre-recorded audio messages and questions using their mobile phone [6]. IVRs have been implemented in the HIC since the 1970’s and may involve call-ins (where users contact a system), call outs (where a system contacts targeted users) or a combination of systems [6, 7]. IVR call-outs, using random digit dialing (RDD), have recently been used in LMICs with mixed results, particularly around representativeness. In Bangladesh and Tanzania, for example, large scale population mobile phone surveys found that IVRs tended to reach younger, male participants of higher education than the national censuses, portraying potential bias toward representing more socio-economically advantaged populations [8].

There are two main types of bias, coverage and non-response, in conducting mobile phone surveys which may contribute to non-representative estimates. Coverage bias is prevalent in populations where mobile phone ownership is not uniform. In 2022, mobile phone ownership in Uganda stood at 79.3% and 89.2% and 74.6% for urban and rural households, respectively [9], meaning mobile phone surveys may exclude up to 21% of the population. Coverage bias could be minimized by equitably reaching urban and rural population in a survey sample. There are few interventions that can remedy coverage bias; for example, requesting phone owners to pass their mobiles to eligible non-phone owners for participation has shown promise [10].

Non-response is important for research because sociodemographic differences between survey participants and those that did not participate, contributes to non-representative surveys, thereby introducing non-response errors or bias to the study outcomes [7, 11–13]. Understanding reasons for non-response could allow for possible interventions to improve response rate and minimize potential biases in surveys. Several interventions have been found to increase IVR survey participation in LMICs. These include user testing to explore participants’ expectations [14–16], cultural adaptation, such as through the use of appropriate language [14, 17], providing airtime incentives for completing surveys [18, 19], and matching the interviewer gender and message valence that would help put the participants at ease [20]. Additionally, deploying shorter surveys (typically less than 10 minutes) [21, 22], sending surveys during evening hours, and ensuring privacy and confidentiality especially within care programs [23, 24] have been found to improve IVR survey participation. Still, non-response for random digit dialed IVR surveys remains high. A study conducted in five LMICs (Philippines, Morocco, Malawi, Sri Lanka and Zambia), found that IVR survey response rates to be between 0.5% and 3.3% [25], consistent with other IVR studies [5, 23], but significantly lower response rates compared to routine face-to-face surveys. However, the growing phone ownership in LMICs continues to provide opportunity for conducting remote, low-cost surveys e.g. through IVR, SMS and CATI, but the low response rates for IVR suggest that large population samples are required for sampling efficiency.

This study aimed to address the knowledge gap on reasons for non-response, and dropping off an IVR survey within the Uganda setting, and to explore how to improve response rates to IVRs using the CATI.

## MATERIALS AND METHODS

### Study design and participants

We conducted a mixed methods study, using CATI (with embedded qualitative questions), followed by IVR, which were nested within an initial IVR mobile phone survey. For the initial IVR survey, RDD was used to generate a sampling frame of Ugandan mobile phone numbers. In MS Excel, the prefixes of all Ugandan mobile network operators were compiled and the remaining digits were randomly generated to create a sampling frame of 10-digit mobile phone numbers. These random phone numbers were uploaded to a third-party’s mobile phone survey platform, ENGAGESpark, which was used to deliver IVR and CATI surveys. Participants who indicated being 18 years or older had access to a mobile phone and spoke one of the four survey languages (Luganda, Luo, Runyakitara, English) were eligible.

### Procedures

IVR surveys were sent using RDD sampling (IVR-RDD) and were delivered from 9 am to 5 pm local time. Potential participants were called up to three times. If participants did not answer the first call, second and third attempts were made 5 minutes later and 2 hours and 55 minutes later, respectively. The last (third) IVR-RDD caller was designed for delivery around 8 pm local time to take advantage of the prior finding of preference for evening calls in the local setting [23, 24]. The IVR-RDD survey contained demographic questions (age, sex, highest level of education and location) as well as tobacco, alcohol and diet questions extracted from WHO STEPS questionnaire. During the survey introduction, participants were told that they would receive an airtime incentive worth 5000 Ugandan Shillings (US$1.3) upon survey completion.

**Figure 1:**
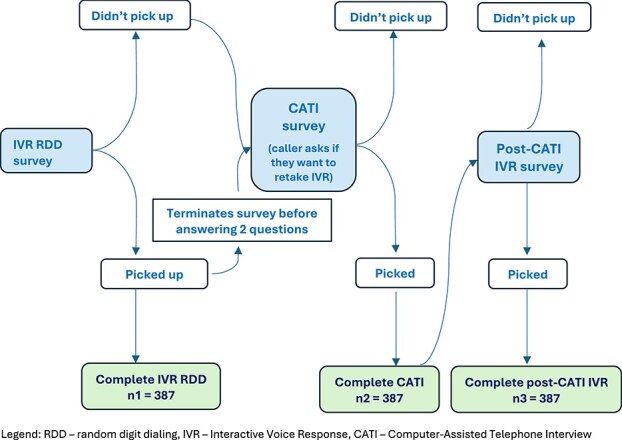
Flowchart of mixed-method IVR-CATI study design assessing non-response, call drop-off and potential for improving response rate.

From the IVR-RDD, participants that did not pick up their mobile phone or who terminated the survey before answering at least two survey questions were called back by the call center stationed at Makerere University and administered a CATI to assess reasons for their non-response (Fig. 1). The call center was staffed by six Ugandans with at least a Bachelor’s level degree. Two call center agents were fluent in each of the three local languages. Call center staff were supervised throughout data collection by a Ugandan supervisor. Daily monitoring of call logs was reviewed, and queries assessed in a weekly meeting.

We hypothesized that by interacting with a human, rather than only through IVR, this may build respondent trust in the survey and therefore higher completion rates upon retaking the IVR survey.

The interviewer guide for the CATI agent had instructions for the caller on how to build rapport with the participants, through a standardized checklist of talking points for frequently asked questions. Frequently asked question answered queries such as (i) ‘how did you get my number?’ (ii) ‘I have already answered a phone survey, why are you calling me?’ (iii) ‘how does this study benefit me, my community or country?’ (iv) ‘I am concerned about security or being conned out of money’, (v) ‘I completed the survey already, and I am waiting for my UGX 5000 (US$1.3) incentive’. After building rapport and answering any participant queries, the interview guide checked the language preference of respondent, asked about prior exposure to a mobile phone survey, then probed on the primary (main/most important) reason for the choice of not participating in the mobile phone survey, followed by exploring the secondary and other or less important reasons for choosing not to participate. Discursive probing was elicited to guide comprehension of the participants’ reasons and thus categorization of reasons. CATI interviews typically lasted between 5 and 8 minutes. After providing reasons for non-response, participants were then asked if they would like to try and complete the IVR survey again (Post-CATI IVR is used to refer to this survey and its respondents). For those who consented to retake the IVR survey, participants were automatically sent the Post-CATI IVR 5 minutes later. This survey was identical to the IVR-RDD survey except it did not include questions on age and sex as this was collected during the CATI. Like the IVR-RDD survey, participants who completed the Post-CATI IVR received 5000 Ugandan Shillings worth of airtime.

### Data management and analysis

For sample size calculation, based on previous research in Uganda, Tanzania, Bangladesh and Colombia on IVR survey completion rates [18, 19], the IVR-RDD was powered at 80%, with 5% margin of error to detect at least 387 complete interviews after the first call, where 20 000 IVR-RDD calls would be send out to achieve the estimated complete IVRs. To ensure quality, validity and ethical reporting, we used the American Association for Public Opinion Research (AAPOR) guidelines [26] to structure the data analysis and reporting of findings from the surveys. AAPOR disposition codes for each call outcomes were defined as follows. First, ineligibles were defined as those who were underage, did not speak language one of the four survey languages, or if the phone number did not exist (was not active). Second, definitions among the eligibles were as follows: Complete interview = I (eligible cases that complete 100% questions in the survey), Partial interview = P (age-eligible cases that answer tobacco questions but dropped off before completing), Refusal and break offs = R (either did not answer consent question for IVR-RDD or were age-eligible but did not complete the tobacco questions), Unknown = U (either hung-up before answering age question or did not pick up the phone), Non-contact = NC (age-eligible participants from the IVR-RDD who did not pick-up the Post-CATI IVR survey) [27]. Following this, the survey rates were computed with the following equations adapted from AAPOR: (a) Contact Rate 1 = (I + P + R)/(I + P + R + NC + U), (b) Response Rate 2 = (I + P)/(I + P + R + NC + U) and (c) Cooperation Rate 1 = I/(I + P + R) [27]. For the Post-CATI IVR, the survey rates were calculated three different ways. In the first model, the calculation was restricted only to phone calls made to deliver the Post-CATI IVR survey. In the second model, disposition codes from the CATI and Post-CATI IVR were included in the calculation. In the third model, disposition codes from all three survey samples were included in the calculation.

By each survey sample (IVR-RDD, CATI and post-CATI IVR), descriptive analyses were generated with counts and corresponding proportions by socio-demographic characteristics. A logistic regression analysis was conducted to assess differences in socio-demographics characteristics for complete interviews between IVR-RDD and Post-CATI IVR. Separately, a logistic regression model was then run to compare key survey rates between the post-CATI IVR and IVR-RDD survey. Contact, response and cooperation rates were used from the third calculation for Post-CATI IVR.

In supplemental analyses, we compared differences in NCD risk factor indicators between the IVR-RDD and post-CATI IVR survey samples using crude and adjusted logistic regression analyses.

For CATI survey participants, the reasons for non-response or drop off were explored through a thematic analysis and reported descriptively, with frequencies and proportions. Subsequently, the findings are stratified by the IVR-RDD subgroups (did not pick up the phone and did not complete the survey) to explore variability, and reported in accordance with the AAPOR definitions.

Analyses were conducted using Stata/SE (version 14.1; Stata Corp, College Station, TX, USA) [28]. An alpha of 0.05 was assumed for all tests of statistical significance. Sample sizes were not inflated for multiple comparisons, which is recommended by Rothman (1990) [29] because of the concern that reducing type I error for a null association in empirical studies increases the type II error probabilities for non-null associations, and thus increasing the sample size was not needed.

### Ethics approvals

Institutional ethics approvals were provided by the Makerere University School of Public Health Research and Ethics Committee (Ref: SPHREC protocol version 445/10/2024) and the Uganda National Council for Science and Technology (UNCST ref: IS 128). The research was conducted in accordance with the principles of the Helsinki declaration [30].

## RESULTS

### Call outcome characteristics by mobile phone survey modality

Overall, 23 288, 9740 and 1000 calls were made for IVR-RDD, CATI and Post-CATI IVR, respectively; yielding 436 (1.9%), 1145 (11.8%) and 449 (44.9%) complete interviews (Table 1). Approximately 81% of IVR-RDD calls were not picked up by a respondent, compared to 6.5% in the Post-CATI IVR sample. An additional 15% (3559/23288) of IVR-RDD calls picked up the phone but hung up before establishing their age eligibility. Refusals were not markedly different between the IVR-RDD and Post-CATI IVR samples. Approximately 87.3% of complete CATIs agreed to retake the IVR survey.

**Table 1:** Call outcome by survey type

	IVR-RDD			CATI			POST-CATI IVR		
Disposition code/call outcome variable	N	%	95%CI	N	%	95%CI	N	%	
Complete interview	436	1.87	(1.70, 2.05)	1145	11.8	(11.1, 12.4)	449	44.9	(41.8, 48.0)
Partial interview	82	0.35	(0.28, 0.44)	9	0.09	(0.04, 0.18)	64	6.40	(5.0, 8.1)
Break-off	74	0.32	(0.25, 0.40)	NA	NA	NA	422	42.2	(39.1, 45.3)
Refusal	77	0.33	(0.26, 0.41)	76	0.78	(0.62, 0.98)	0	0	(0.00, 0.37)
Unknown: picked up phone	3559	15.3	(14.8, 15.6)	1860	19.1	(18.3, 19.9)	NA	NA	NA
Unknown: did not pick up phone	18 837	80.9	(80.4, 81.4)	6292	64.6	(63.6, 65.6)	NA	NA	NA
Non-contact: eligible did not pick up phone	NA	NA	NA	NA	NA	NA	65	6.5	(5.05, 8.21)
Ineligible: under 18 years old	226	0.97	(0.85, 1.10)	2	0.02	(0.00, 0.07)	NA	NA	NA
Ineligible: phone number not active	NA	NA	NA	295	3.03	(2.70, 3.39)	NA	NA	NA
Ineligible: Does not speak language	NA	NA	NA	61	0.63	(0.48, 0.80)	NA	NA	NA
**Total calls made**	**23 288**	**100**		**9740**	**100**		**1000**	**100**	
**Wants to take POST-CATI IVR survey**				**1000/1145**	**87.3**				

Abbreviation: POST-CATI IVR, IVR sent after consenting during CATI.

### CATI method exploring reasons for IVR-RDD survey non-participation

Approximately 65% (6292/9740) of the participants who did not pick up the IVR-RDD and were called back by the call center did not pick up the phone to initiate the CATI (Table 1). The platform log of reasons for non-response included: (i) phone rang but did not pick up 89% (5605/6292), (ii) phone switched off by user 6% (363/6292) and (iii) phone connection failed 4% (236/6292; Table S1).

Participants indicated that being busy at the time of receiving the IVR was the most common reason for IVR-RDD non-response among those who either did not pick up their phone (45.2%; 198/438) or did not complete the survey (43%; 332/773, Table 2). For those that did not pick up the phone, not hearing the phone ring was the second largest reason (42.7%; 187/438). For those that did not complete the survey, not understanding the instructions was the second most common reason (25.4%; 196/773) Other prevalent reasons, though individually accounting for just under 5% of reasons included: a non-recognition of the caller, network coverage drops, thought it was spam and phone operation challenges.

**Table 2:** Primary reason for not answering the sent interactive voice response survey (IVR-RDD) by whether participant initiated the survey

	Did not pick up phone	95%CI	Did not complete survey	95%CI
Was too busy at time of call	198 (45.2%)	(40.5, 50.0)	332 (43.0%)	(39.4, 46.5)
Did not hear phone ring	187 (42.7%)	(38.0, 47.5)	97 (12.6%)	(10.3, 15.1)
Did not understand the instructions	7 (1.60%)	(0.64, 3.27)	196 (25.4%)	(22.3, 28.6)
Did not recognize the phone number	18 (4.11%)	(2.45, 6.42)	26 (3.36%)	(2.21, 4.86)
Network coverage dropped	6 (1.37%)	(0.50, 2.96)	35 (4.53%)	(3.17, 6.24)
Though it was spam	5 (1.14%)	(0.37, 2.64)	21 (2.72%)	(1.69, 4.12)
Battery died	4 (0.91%)	(0.25, 2.32)	5 (0.65%)	(0.21, 1.50)
Was not interested	6 (1.37%)	(0.50, 2.96)	5 (0.65%)	(0.21, 1.50)
Preferred not to participate in phone surveys	0 (0%)	(0.00, 0.84)	4 (0.52%)	(0.14, 1.32)
Operating phone challenges	2 (0.46%)	(0.06, 1.64)	36 (4.66%)	(3.28, 6.39)
Poor sound quality/Inaudible	0 (0%)	(0.00, 0.84)	6 (0.78%)	(0.29, 1.68)
Other	5 (1.14%)	(0.37, 2.64)	10 (1.29%)	(0.62, 2.37)
**Total**	**438**		**773**	

Data are n (%) and come from complete and partial interviews administered by a call center agent.

### Characteristics of participants with complete interviews

Table 3 shows the sociodemographic characteristics of complete interviews by survey type. A significantly higher proportion of females responded to a post-CATI IVR (37%; 167/449) compared to the initial IVR-RDD (26%; 112/436); OR 1.71, 95%CI 1.28, 2.28, *P* < 0.001. Post-CATI IVR also had significantly higher distributions of those 30–44 years (OR = 1.73 95%CI: 1.30, 2.32), 45–59 years (OR = 2.45 95%CI: 1.42, 4.24) and 60 and above (OR = 3.12 95%CI: 1.27, 7.66), as compared to IVR-RDD. When stratified by sex, post-CATI IVR had significantly increased representation of the older age groups in males, compared to IVR-RDD, though this was not significant in females aged 30–44 years (OR = 1.59 95%CI: 0.95, 2.68, *P* = 0.077) and 45–59 years (OR = 3.45 95%CI = 0.94, 12.7, *P* = 0.062). Overall, the gains in female and older age groups of the Post-CATI IVR sample were less than the gains in the CATI sample, as compared to IVR-RDD.

**Table 3:** Sociodemographic characteristics of complete interviews and their associations by survey type

	IVR-RDD		CATI		POST-CATI IVR			
Characteristic	n	%	n	%	n	%	Odds ratio (95%CI)	*P*-value
**Sex**								
Male	324	74.3%	626	54.7%	282	62.8%	Ref.	
Female	112	25.7%	519	45.3%	167	37.2%	1.71 (1.28, 2.28)	<0.001
**Age group**								
18–29 years	279	64.0%	424	37.0%	217	48.3%	Ref.	
30–44 years	128	29.4%	453	39.6%	173	38.5%	1.73 (1.30, 2.32)	<0.001
45–59 years	22	5.1%	178	15.5%	42	9.4%	2.45 (1.42, 4.24)	0.001
60+ years	7	1.6%	90	7.9%	17	3.8%	3.12 (1.27, 7.66)	0.013
**Male-age group**								
18–29 years	204	63.0%	217	34.7%	130	46.1%	Ref.	
30–44 years	94	29.0%	246	39.3%	110	39.0%	1.83 (1.29, 2.61)	0.001
45–59 years	19	5.9%	113	18.0%	30	10.6%	2.48 (1.34, 4.58)	0.004
60+ years	7	2.2%	50	8.0%	12	4.3%	2.69 (1.03, 7.01)	0.043
**Female-age group**								
18–29 years	75	67.0%	207	39.9%	87	52.1%	Ref.	
30–44 years	34	30.4%	207	39.9%	63	37.7%	1.59 (0.95, 2.68)	0.077
45–59 years	3	2.7%	65	12.5%	12	7.2%	3.45 (0.94, 12.7)	0.062
60+ years	0	0%	40	7.7%	5	3.0%	Not estimable	
**Education**								
None	56	12.8%	174	15.9%	41	9.7%	Ref.	
Primary	135	31.0%	302	27.6%	105	24.8%	1.06 (0.66, 1.71)	0.804
O level	122	28.0%	339	31.0%	139	32.9%	1.56 (0.97, 2.49)	0.065
A level	44	10.1%	132	12.1%	60	14.2%	1.86 (1.06, 3.26)	0.030
University/post-grad	79	18.1%	147	13.4%	78	18.4%	1.35 (0.81, 2.25)	0.251
Other			*n* = 51		*n* = 26			
**Location**								
Urban	214	49.1%	NA		218	48.6%	Ref.	
Rural	222	50.9%	NA		231	51.5%	1.01 (0.89, 1.15)	0.875

Abbreviation: POST-CATI IVR, IVR sent after consenting during CATI. Odds ratio compares POST-CATI IVR with IVR-RDD (reference).

### Changes to survey rates by study arm

Table 4 presents the contact, response and cooperation rate by survey type. Contact and response rates, respectively, were higher for Post-CATI IVR (model 1; 93.5% and 51.3%) as compared to IVR-RDD (2.90 and 2.25). Contact and response rates for Post-CATI IVR decreased when including disposition codes from the previous survey sample in their analysis. The final response and survey rates for Post-CATI IVR were 7.74% and 4.54%, respectively. Specifically, the contact rate for post CATI IVR had nearly three-fold higher odds than for the IVR-RDD (OR 2.81, 95%CI 2.56, 3.08, *P* < 0.001). Similarly, the post-CATI response rate was about two-fold higher than that of the IVR-RDD (OR 2.07, 95%CI 1.86, 2.30, *P* < 0.001). On the other hand, the cooperation rate significantly declined between the Post-CATI IVR (50.3%; 885/1758) and IVR-RDD (65.2%; 436/669); OR 0.54, 95%CI 0.45, 0.65, *P* < 0.001.

**Table 4:** Key survey rates by study arm

	IVR-RDD	CATI	POST-CATI IVR^1^	POST-CATI IVR^2^	POST-CATI IVR^3^	Odds ratio (95%CI)	*P*-value
Contact rate #1	669/23062 (2.90)	1231/9383 (13.1)	935/1000 (93.5)	1089/9383 (11.6)	1758/22704 (7.74)	2.81 (2.56, 3.08)	<0.001
Response rate #2	518/23062 (2.25)	1155/9383 (12.3)	513/1000 (51.3)	513/9383 (5.47)	1031/22704 (4.54)	2.07 (1.86, 2.30)	<0.001
Cooperation rate #1	436/669 (65.2)	1146/1231 (93.1)	449/935 (48.0)	449/1089 (41.2)	885/1758 (50.3)	0.54 (0.45, 0.65)	<0.001
Average time per complete survey	7 min 32 sec		4 min 30 sec				
No. of calls per complete survey	53.4	8.5	2.2	21.7	26.3		

Survey rates are n/N (%) and are defined by American Association of Public Opinion Research. ^1^Model 1 was restricted only to disposition codes from Post-CATI IVR. ^2^Model 2 includes disposition codes from model 1 and disposition codes from CATI participants. ^3^Model 3 includes disposition codes from model 2 and disposition codes from IVR-RDD participants. Odds Ratio is comparison of model 3 POST-CATI IVR and IVR-RDD

The average time to complete the post-CATI IVR was four-and a-half minutes, unlike the IVR-RDD that lasted nearly seven and a half minutes (Table 4). There were no differences in NCD risk indicators between the IVR-RDD and Post-CATI IVR (Table S2).

## DISCUSSION

This study aimed to assess the reasons for non-response to IVR surveys once initiated and to assess whether a brief interaction with a human call center agent could lead to subsequent gains in response and completion rates. We found that those who spoke with a call center agent were significantly more likely to complete the follow-up IVR survey. Survey completion rates were 1.9% in our IVR-RDD, showing low IVR completion, but comparable to other LMICs where IVR response ranges between 0.5% and 3.3% [25]. CATI completion was 11.8% comparable to 15% in Nigeria [31], while post-CATI response rates in this study at 44.9% are comparable to direct CATI completion in Burkina Fasso at 49.8% [32], although CATI is resource-intense in relation to IVR. Understanding reasons for non-response may inform future interventions or modifications to the IVR survey to improve survey participation.

The most common reason for not completing or not picking-up the initial IVR-RDD survey was that the respondents indicated that they were too busy, which is empirically reflected in the low response and cooperation rates found in IVR-RDD sample. Interestingly, the response rate and cooperation rate of CATI sample were significantly higher than the IVR-RDD sample which suggests that this opinion may be tempered by the addition of a call center agent, or in other words, the interaction with a human being [31]. This notion is further supported by the lower observed cooperation rate, or the proportion of complete interviews from eligible participants observed in the post-CATI IVR sample. Previous studies from Bangladesh, Colombia and Tanzania have found that CATI yields higher response and cooperation rates than IVR surveys of NCD risk factors [10]. This has also been observed in other survey topics; in Nigeria CATI had five-fold higher response rates compared to IVR [31], and in Burkina Faso, CATI surveys had higher response rates than did IVR surveys. This approach of employing a call center agent to call back participants who did not complete an IVR survey, a mixed-mode approach, may be an alternative to improving survey representativeness. Additional costing analyses are needed [7, 31].

Where introducing a call center agent may not be feasible due to financial and personal resource constraints, altering the timing of the IVR delivery or even providing the opportunity to schedule an IVR may counteract the identified challenge of being too busy. Optimizing the time window when most participants might be less busy with economic-related activities, as some studies in varied settings have suggested preference for times such as 6 pm to 10 pm in Uganda, and after workhours for Bangladesh [21, 22] may see gains in survey participation. Qualitative exploration of willingness for potential users to engage with IVRs is important [33], as it enhances community engagement, although the limitation for IVRs as with other surveys is that it is limited to a specific goal e.g. NCD screening, or enhancing continuity of diabetes care [34].

The second most prevalent reason for non-completion of the IVR survey, was a lack of understanding of IVR instructions leading to dropping off during the IVR-RDD for one-quarter of callers. This finding suggests a limited engagement of participants with the IVR phone interface, or a low literacy level emphasizing a thorough pre-testing, cultural adaptation and pre-sensitization of intended users. Nonetheless, language and technological literacy are highly prevalent in LMICs [12, 19, 21] and are difficult to circumvent so IVR innovators need to achieve user ease, and customer friendliness through the IVR engagement [34].

A third reason for non-response to the IVR was related to trust and/or a lack of connection with the caller identity, about 1 in 20 participants in both groups (did not pick up and did not complete survey) reported that they either did not identify the caller or thought it was spam. There is need to enhance credibility for IVR and online surveys, particularly those where respondents were not expecting a survey, like in RDD. This finding emphasizes a need for pre-engagement, or for the creation of a feedback loop into the IVR system to permit further engagement for building trust between the provider and user. Mistrust from users of the IVR providers has been reported before in Uganda and Bangladesh [21, 22], highlighting a need for continuous engagement with potential IVR users. As noted in care delivery models, the promise of privacy and confidentiality increases trust and engagement such as for cancer, fistula and HIV/AIDs care [23, 24, 35]. Although our IVR survey indicated that the survey was sponsored by Makerere University, more effort may be needed. Others have found that sending a pre-survey text message notification with details about the upcoming survey, including the sponsor, significantly improves phone survey response rates [36].

Additionally, about 1 in 20 participants among the non-completes of the IVR survey reported phone operation challenges which might reflect a non-familiarity with mobile phone technology. This challenge is expected to self-regress over time as mobile phone technology becomes more diffuse among the population, but as discussed earlier, language and technology literacy are limitations to technological adaptation. Relatedly, only 1 in 20 participants among the non-completes for the IVR survey reported network connectivity challenges, implying that connectivity in urban and rural areas of Uganda has improved over time. Minor reasons for dropping off related to technological issues such as sound inaudibility, poor battery and notably <2% of participants declined to participate, offering promise of interest to participate in IVR surveys if they are well designed and executed among potential users.

Regarding population-level assessment of NCD risk factors, the IVR-RDD and post CATI IVR had comparable results showing that they are both valid methods with comparable psychometric properties. This implies that for the assessment of risk factors for NCDs, the initial IVR-RDD is a sufficient screening tool, and there is no added specificity or sensitivity through potential enhancement of method from a follow-on expensive CATI and a post-CATI IVR. This finding supports the growing evidence on cross-method validation of mobile phone survey methodologies in relation to NCD risk factor estimation [28, 37].

This study has notable strengths. As the IVR-RDD was implemented to a random sample of mobile phone owners, while the CATI was implemented to a sample of all non-responders (did not pick, or terminated call early) yielding 87.3% reach, this minimized selection bias. Further, the post-CATI IVR was offered to all consenting persons at the CATI enhancing random selection among previous IVR-RDD non-responders and increasing overall IVR response. IVR-RDD caller attempts were also delivered until 8 pm local time, enhancing potential reach as prior local IVR research had indicated user preference for evening hours. Finally, a mixed method approach supported a quick exploration of reasons for non-response, while simultaneously attempting to increase response rates.

Our study has some limitations as well. First, we primarily used quantitative approaches to infer qualitative data, essentially taking a positivist ontological perspective. The mixed method techniques, the IVR-RDD (quantitative), CATI (embedded qualitative questions) and post CATI IVR (quantitative) were used to collectively assess reasons for non-response to IVR and explore potential for improving non-response to IVRs. While the method had a wide reach of more than 30 000 participants, technically, a small sample, such as focus group discussions for in-depth exploration of reasons for non-response could have yielded additional reasons. As such, the study was limited in breadth by design to explore a plethora of potential reasons for non-response. Embedded qualitative narrative interviews within IVR surveys is recommended to augment recruitment efforts for IVR survey populations, and to enhance IVR survey experience.

The tools of potential reasons for drop-off were developed a priori, based on researchers’ previous experience with the IVR platform, and qualitative findings from exploratory work in various contexts. A pre-testing phase was conducted as part of research assistant training. However, the study team cannot claim an exhaustive theoretical framework development for reasons for non-response and drop-off, rather the value of the study lies in the validation of some reasons for non-response and drop-off constructs that are derived from the existing body of knowledge. In so doing, this contributes to theory generation on reasons for non-response to IVR. Further research is needed, both inductive and deductive.

Also, the Ugandan context is multi-lingual whereby the IVR survey used only four predominant languages which could have inadvertently led to misrepresentation of ethnic minorities. However, the three predominant local dialects: Luganda, Runyakitara and Luo in addition to English (the official language) are expected to reach ~80% of the local population, thereby offering reasonable representation.

Finally, this study could not entirely eliminate selection and coverage bias. As noted prior, selection bias relates to whether participant (responders’) characteristics may defer from non-participants. In this study, 87.3% (1000/1145) CATI participants accepted a post-CATI survey, but it was not possible to compare these with the 12.7% (*n* = 145) that declined the post-CATI IVR. On the other hand, coverage bias relates to under- and over- representation of population groups based on attributes such as mobile phone ownership. Therefore, given the mobile phone ownership in Uganda at 79.3% [9], coverage bias could have been introduced by the omission of nearly 21% population who do not own phones [10].

## CONCLUSION

This study found the main reasons for non-response and dropping off an IVR-RDD exploring NCD risk factors to include being busy, lack of understanding of IVR instructions, and a lack of trust and connection to the caller/IVR provider. This affords opportunities to tailor interventions to improve future survey participation. Technical issues such as phone operation challenges or network connectivity were seldom reported. In addition to providing a venue to collect reasons for non-response, the post-CATI IVR had marked improvement in contact and response rates as compared to the initial IVR; demonstrating that embedding CATI surveys within IVRs has potential to improve demographic representativeness and response rates to the IVR-RDD.

Where feasible, post-CATI IVR samples could be used for routine monitoring or even validating hypothesized reasons for non-response or high drop-offs from IVRs to enhance the IVR design, delivery timing and other IVR attributes that may improve contact, response and cooperation rates. Still, there is a need to improve the appeal of IVRs among the target population so that it becomes a priority—either through prior sensitization/mobilization or enhancing caller credibility, as well as targeting IVRs when people are less busy.

## Supplementary Material

Appendix_Tables_Reasons_phone_not_ans_called_by_a_human_enumerator_oqaf017

Supplementary_materials_oqaf017

## Data Availability

The datasets generated or analyzed during this study are available upon request to the corresponding author. Additional data are available on the data for health resource library at https://data4healthlibrary.org/countries/uganda.
