# Supplementary material for: Exploring barriers to IVR surveys and the effectiveness of human follow-up calls: insights from a mixed methods study in Uganda
Source: Oxf Open Digit Health. 2025 Aug 16;3:oqaf017. doi: 10.1093/oodh/oqaf017 (PMC12413760; doi:10.1093/oodh/oqaf017)
Supplement: Appendix_Tables_Reasons_phone_not_ans_called_by_a_human_enumerator_oqaf017 [file appendix_tables_reasons_phone_not_ans_called_by_a_human_enumerator_oqaf017.docx]

**Appendix**

**Table S1. Reasons phone not answered when called by a human enumerator (CATI)**

|  | N | % | 95%CI |
| --- | --- | --- | --- |
| Phone connection failed | 236 | 3.75% | (3.30, 42.50) |
| Phone is busy | 88 | 1.40% | (1.12, 1.72)) |
| Phone switched off | 363 | 5.77% | (5.21, 6.37) |
| Phone rang, but did not pick up | 5605 | 89.08% | (88.3, 89.8) |
| **Total** | 6292 | 100.0% |  |

**Table S2.** **Noncommunicable disease risk factor estimates for complete interviews by survey type**

| **Indicator** | **IVR-RDD** | **POST-CATI IVR** | **Crude OR (95%CI)** | **p-value** | **Adj. OR (95%CI)** | **p-value** |
| --- | --- | --- | --- | --- | --- | --- |
| Current smoker | 44 (10.1%) | 44 (9.8%) | 0.97 (0.62, 1.50) | 0.885 | 1.20 (0.75, 1.92) | 0.439 |
| Daily smoker | 25 (5.7%) | 18 (4.0%) | 0.69 (0.37, 1.28) | 0.235 | 0.93 (0.48, 1.79) | 0.832 |
| Former smoker | 50 (11.5%) | 50 (11.1%) | 0.97 (0.64, 1.47) | 0.876 | 1.00 (0.65, 1.57) | 0.979 |
| Current smokeless user | 71 (16.3%) | 55 (12.2%) | 0.72 (0.49, 1.05) | 0.087 | 0.78 (0.52, 1.17) | 0.233 |
| Daily smokeless user | 40 (9.2%) | 24 (5.3%) | 0.56 (0.33, 0.94) | 0.030 | 0.64 (0.37, 1.11) | 0.115 |
| Former smokeless user | 47 (10.8%) | 43 (9.6%) | 0.88 (0.57, 1.36) | 0.554 | 0.94 (0.59, 1.50) | 0.800 |
| 1 year alcohol abstinence | 237 (54.4%) | 235 (52.3%) | 0.92 (0.71, 1.20) | 0.547 | 0.90 (0.68, 1.19) | 0.462 |
| Current drinker (30 days) | 121 (27.8%) | 125 (27.8%) | 1.00 (0.75, 1.35) | 0.977 | 1.02 (0.75, 1.40) | 0.870 |
| Mean fruit days* | 3.45 (0.10) | 3.82 (0.11) | 0.37 (0.07, 0.67) | 0.014 | 0.28 (-0.03, 0.59) | 0.075 |
| Mean fruit servings* | 1.78 (0.11) | 1.92 (0.12) | 0.14 (-0.17, 0.45) | 0.371 | 0.16 (-0.16, 0.49) | 0.319 |
| Mean vegetable days* | 3.30 (0.09) | 3.28 (0.09) | -0.02 (-0.28, 0.24) | 0.881 | -0.11 (-0.38, 0.16) | 0.441 |
| Mean vegetable servings* | 1.89 (0.13) | 1.92 (0.14) | 0.03 (-0.35, 0.40) | 0.884 | 0.01 (-0.38, 0.40) | 0.963 |
| <5 fruit & veg serving/day | 340 (78.0%) | 340 (75.7%) | 0.88 (0.64, 1.20) | 0.426 | 0.87 (0.63, 1.22) | 0.424 |
| Ever had BP measured | 144 (33.0%) | 199 (44.3%) | 1.61 (1.23, 2.12) | 0.001 | 1.28 (0.95, 1.72) | 0.108 |
| Hypertension diagnosis | 77 (17.7%) | 103 (22.9%) | 1.39 (0.99, 1.93) | 0.052 | 1.12 (0.78, 1.60) | 0.531 |

Abbreviations: IVR, interactive voice response; RDD, random digit dialing; POST-CATI IVR, second attempt at IVR survey; RR, risk ratio. Data are n (%) unless otherwise indicated. Logistic regressions were used to estimate odds ratio and corresponding 95%CI. Adjusted models include sex, age, education, and location. *Data are n (standard error)
